# A Qualitative Study of Patient and Healthcare Provider Perspectives on Building Multiphasic Exercise Prehabilitation into the Surgical Care Pathway for Head and Neck Cancer

**DOI:** 10.3390/curroncol29080469

**Published:** 2022-08-21

**Authors:** Julia T. Daun, Rosie Twomey, Joseph C. Dort, Lauren C. Capozzi, Trafford Crump, George J. Francis, T. Wayne Matthews, Shamir P. Chandarana, Robert D. Hart, Christiaan Schrag, Jennifer Matthews, C. David McKenzie, Harold Lau, S. Nicole Culos-Reed

**Affiliations:** 1 Faculty of Kinesiology, University of Calgary, Calgary, AB T2N 1N4, Canada; 2 Ohlson Research Initiative, Arnie Charbonneau Cancer Institute, Cumming School of Medicine, University of Calgary, Calgary, AB T2N 4N1, Canada; 3 Department of Community Health Sciences, Cumming School of Medicine, University of Calgary, Calgary, AB T2N 4N1, Canada; 4 Section of Otolaryngology Head & Neck Surgery, Department of Surgery, Cumming School of Medicine, University of Calgary, Calgary, AB T2N 4N1, Canada; 5 Foothills Medical Centre, Alberta Health Services, Calgary, AB T2N 2T9, Canada; 6 Department of Clinical Neurosciences, Cumming School of Medicine, University of Calgary, Calgary, AB T2N 4N1, Canada; 7 Department of Surgery, Cumming School of Medicine, University of Calgary, Calgary, AB T2N 4N1, Canada; 8 Department of Oncology, Cumming School of Medicine, University of Calgary, Calgary, AB T2N 4N1, Canada; 9 Section of Plastic and Reconstructive Surgery, Department of Surgery, University of Calgary, Calgary, AB T2N 4N1, Canada; 10 Department of Psychosocial Resources, Tom Baker Cancer Centre, Cancer Care, Alberta Health Services, Calgary, AB T2N 4N2, Canada

**Keywords:** head and neck cancer, exercise oncology, prehabilitation, patient-oriented research, cancer survivorship, qualitative research

## Abstract

Head and neck cancer (HNC) surgical patients experience a high symptom burden. Multiphasic exercise prehabilitation has the potential to improve patient outcomes, and to implement it into the care pathway, the perspectives of patients and healthcare providers (HCPs) must be considered. The purpose of this study was thus to gather feedback from HNC surgical patients and HCPs on building exercise into the standard HNC surgical care pathway. *Methods:* Semi-structured interviews were conducted with patients and HCPs as part of a feasibility study assessing patient-reported outcomes, physical function, and in-hospital mobilization. Interview questions included satisfaction with study recruitment, assessment completion, impact on clinical workflow (HCPs), and perceptions of a future multiphasic exercise prehabilitation program. This study followed an interpretive description methodology. *Results:* Ten patients and ten HCPs participated in this study. Four themes were identified: (1) acceptability and necessity of assessments, (2) the value of exercise, (3) the components of an ideal exercise program, and (4) factors to support implementation. *Conclusion:* These findings highlight the value of exercise across the HNC surgical timeline from both the patient and the HCP perspective. Results have informed the implementation of a multiphasic exercise prehabilitation trial in HNC surgical patients.

## 1. Introduction

Over 550,000 people worldwide are diagnosed with head and neck cancer (HNC) each year [1,2]. HNC surgical patients experience high symptom burden from treatment, which can impact their physical (e.g., neck and shoulder mobility, speech, and swallowing) and psychosocial functioning (e.g., depression, anxiety, quality of life) [3,4,5,6,7,8]. To improve patient outcomes, multiphasic exercise prehabilitation is a possible addition to the HNC clinical care pathway.

Multiphasic exercise prehabilitation is an intervention typically delivered over the surgical timeline and into recovery to improve peri and post-operative outcomes [9,10,11,12]. While exercise for HNC supports its feasibility and impact for improving patient outcomes after adjuvant treatment (e.g., chemotherapy; radiotherapy), there is little in the literature that examines a multiphasic exercise prehabilitation approach that considers the role of exercise after diagnosis before surgery, after surgery while in immediate recovery (in and out of hospital), and into recovery before and subsequently concurrent to adjuvant treatment. Specifically, multiphasic exercise prehabilitation may help reduce treatment-related impairments or side effects, the risk of post-surgical complications, hospital length of stay, and costs of care [9,10,13,14,15]. While it is increasingly recognized that exercise can be a supportive cancer care therapy that helps counteract the adverse effects of cancer and its treatment into survivorship [16,17], implementing a multiphasic exercise prehabilitation program as part of clinical care for a complex patient population is challenging [16,17,18,19].

In Calgary, Alberta, current peri-operative HNC care already includes several presurgical consultations, multi-site wound care, speech and swallowing rehabilitation, nutritional care, early mobilization via the Enhanced Recovery After Surgery (ERAS) protocol, and post-discharge follow-up appointments [20]. The use of assessments such as patient-reported outcomes (PROs), physical function, and in-hospital mobilization are important to inform the feasibility and impact of multiphasic exercise prehabilitation. Thus, to support a multiphasic exercise prehabilitation approach into this clinical care pathway, gathering perspectives from both patients and healthcare providers (HCPs) is critical. Utilizing a patient-oriented research approach can provide information on exercise barriers and preferences and help identify patient priorities and unmet wellness needs [21,22]. In addition, feedback from HCPs is critical for understanding clinical workflow to support multiphasic exercise prehabilitation implementation [21,22].

The purpose of this qualitative study was therefore to understand patient and HCP perspectives on the role of multiphasic exercise prehabilitation considering unique needs across the surgical timeline for HNC patients. Specifically, feedback was obtained on: (1) adding exercise-related assessments across the HNC surgical timeline and (2) the logistics and potential benefits of a multiphasic exercise prehabilitation intervention.

## 2. Methods

### 2.1. Participants 

Participants included HNC surgical patients and HCPs (those directly involved in patient care, including surgeons, nurses, physiotherapists) who were part of our prospective cohort feasibility study [23]. This feasibility study examined the completion of and satisfaction with implementing PROs (measured via online questionnaires), physical function (measured via in-person assessments), and in-hospital mobilization (measured via a Garmin Vivofit 4 activity tracker) assessments across the HNC surgical timeline [23]. This study was approved by the Health Research Ethics Board of Alberta Cancer Committee (HREBA.CC-18-0564) and both verbal and written consent were documented via a secure web application (Research Electronic Data Capture; REDCap) [24].

### 2.2. Interview Procedures

The lead author conducted semi-structured interviews with patients and HCPs (≤45 min duration) to gather feedback on completing assessments of PROs, physical function, and in-hospital mobilization across the surgical timeline, as well as on the logistics surrounding a future multiphasic exercise prehabilitation intervention, including impact on clinical workflow. These interview guides are available as a Supplementary File S1. In some cases, patient caregivers were also present to support answering questions for patients who had difficulties speaking post-surgery. If necessary, the lead author paraphrased responses, and confirmed perspectives were accurately captured through prompted questions. Interviews were recorded via a study-specific voice recorder.

### 2.3. Interviews with Patients

Patients were invited to take part in an interview at two timepoints: (1) in-hospital, 7–14 days post-surgery, and (2) 6 ± 2 weeks post-surgery, during a follow-up assessment at the University of Calgary. Interviews were optional and took place only if/when the patient was feeling well enough to take part, according to the judgement of HCPs and in addition to verbal consent from the patient. Some patient interviews took place at the start of the COVID-19 pandemic and as such, were conducted remotely, via phone call, instead of in-hospital or at the follow-up in-person assessment. 

Questions during the inpatient interview captured patient satisfaction with completing assessments in the study (e.g., could you please tell me about your overall experience with the study thus far?) and took place in the participant’s private hospital room. The second interview gathered important information on the perceived value of implementing a future multiphasic exercise prehabilitation intervention for HNC surgical patients. Specifically, patients were provided with a multiphasic exercise prehabilitation program scenario and were asked questions about program components (e.g., frequency, intensity, time, and type (FITT) principles, as well as other aspects of the intervention, such as preferred location for exercise). To facilitate patients’ understanding of these exercise principles, prompts such as the Rating of Perceived Exertion scale [25] were used as applicable. For the patients who did not complete the in-hospital interview, the second interview combined both sets of questions.

### 2.4. Interviews with Healthcare Providers

Interviews with HCPs occurred in the last two months of patient recruitment into the feasibility study [23] once clinical workflows had been established. Interviews took place either at the Foothills Medical Centre in Calgary, Alberta or remotely via phone call. Interview questions were designed to obtain feedback on the satisfaction of the recruitment and assessment procedures (e.g., please tell me about your experience with this recruitment process), ease of implementation (e.g., please tell me about what you think about adding in these assessments to the usual care for the HNC surgical patients), and clinical logistics (e.g., what do you need from us, as exercise experts, to make this successful within the hospital setting?). HCPs were also asked about the perceived value of implementing a multiphasic exercise prehabilitation intervention for HNC surgical patients and were provided with the same scenario as patients, in addition to questions specific to the clinical logistics of implementation. Interview guides were modified to ensure questions were asked specific to each HCP role.

### 2.5. Qualitative Methodology, Interpretive Description

This study was guided by an interpretive description methodology [26] and constructivist philosophy [27], wherein articulating the research question, conducting interviews, and analyzing and disseminating data followed their principles. To ensure a rigorous study, the four approaches specific to interpretive description were followed: epistemological integrity, representative credibility, analytic logic, and interpretive authority [26]. For the context of this study, interpretive description was used to inform multiphasic exercise prehabilitation integration within clinical HNC practice.

### 2.6. Analysis

Interviews were transcribed verbatim in Express Scribe [28] and analyzed in NVivo 12 [29] by the lead author. Specifically, the lead author took transcript notes and coded the transcripts in NVivo. Subsequently, themes were generated by three authors, JD, RT, and NCR. Quotes with repetitive words, lip smacks, or mumbled speech were replaced with “[…]” to enhance readability, and identifiable information was removed. Additionally, content experts in interpretive description were consulted throughout the analysis around the interview themes via critical discussion to ensure themes did not overlap nor arise based solely upon the questions asked.

## 3. Results

### 3.1. Participants 

Participant characteristics are presented in Table 1. From the 16 patients who participated in the feasibility study and from the 10 HCPs approached, a total *n* = 20 of patients (*n* = 10) and HCPs (*n* = 10) participated in this qualitative portion. The six patients who did not participate in an interview either declined (*n* = 1), dropped out of the larger feasibility study (*n* = 1), or were not approached by the study team due to the study ending early due to the impact of COVID-19 (*n* = 4). HNC surgical patients ranged in age from 44 to 71 years (mean ± SD; 61 ± 9 years) and were primarily male (*n* = 9). Due to symptom burden (primarily due to fatigue or difficulties speaking), only one patient completed an interview at both timepoints. Nine completed an interview 6 ± 2 weeks post-surgery, of which three took place remotely. Two caregivers were present, one each at two individual interviews. Ten HCPs participated in interviews, four of whom identified as male and six as female. Six of the ten interviews took place remotely. 

### 3.2. Themes

**Figure 1:**
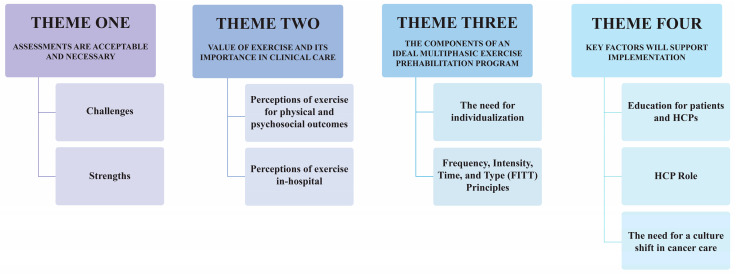
Main themes and sub-themes from interviews.

Four unique themes were identified and are presented in Figure 1. Representative participant quotes (i.e., patient and HCP quotes) are presented below.

#### 3.2.1. Theme One: Assessments Are Acceptable and Necessary

##### Patient Perspectives

All patients found the overall recruitment and assessment process to be manageable, appropriate, and relevant to their experiences across the surgical care pathway. Further, patients noted they were happy to participate in a study that could improve care for future patients. While most patients completed questionnaires, the volume was sometimes viewed as excessive or repetitive (*n* = 7) and some of the individual questionnaire items were not applicable. Having the ability to answer “not applicable” or to skip or streamline questions was identified as important.

*The questionnaires, the only difficulty I had was the very first time and the one in the hospital, some things hadn’t happened yet so it was unclear how to answer those because there was no ‘not applicable’, or something like that or a way I could say ‘I haven’t experienced that problem yet so it doesn’t count’ that was my only real problem with those.*

P 6.

Conversely, other patients found completing the questionnaires allowed them to prepare themselves prior to surgery, reflect on their overall experience, and articulate their recovery. Patients also found the tests of physical function to be relevant to functional changes as a result of surgery and enjoyed participating in these assessments.

*Oh, no problem with that. Yeah, that makes sense to find out how fast one recovers from the surgery. I think is really quite critical and I sort of almost wondered if there would be another one like a week or ten days later, to see how the improvement is.*

P 6.

Finally, while patients perceived the use of the activity tracker (to measure mobility in-hospital) as acceptable, many would have preferred more education surrounding its purpose.

*Hindsight you probably could’ve put a bit more emphasis put on the watch and ‘hey we’re gonna track what you’re doing and really want you to push yourself each day’.*

P 8.

##### HCP Perspectives

HCPs described challenges and strengths with the steps taken to implement exercise into the care pathway for HNC surgical patients. HCPs described the surgical consent process as an overwhelming time for patients, and although patients are receptive to hearing about an opportunity that may enhance their experience, only a quick conversation is practical.

*I think we know it’s a very challenging patient population, not because of the people, particularly but because of what they’re being subjected to […] it’s a time-sensitive cancer…when surgery’s involved is complex and so there’s a lot of moving parts.*

HCP 6—Surgeon.

Other challenges were expressed surrounding the use of the activity tracker (e.g., adding this documentation to standard clinical workflow). However, once workflow was established, the tracker was viewed positively for enhancing patient outcomes and for facilitating the documentation of movement.

*As you may know, we’re not very good at documenting mobility, and hence, why I think that these uhm, [activity trackers], might be very useful for nurses in helping reduce some of their workload.*

HCP 1—Clinical Nurse Educator.

A main strength described by HCPs was the ongoing and clear communication that led to the development of new workflow practices. One HCP felt proud of the new practices to document multiphasic exercise prehabilitation for patients.

*I think we’re all really interested and excited and really proud that this is happening for our patients.*

HCP 8—Surgeon.

HCPs also found the PRO and physical function assessments helped patients articulate their experiences.

*[patient-reported outcomes] get patients to intellectualize what’s going on with their body, and I think that can be a really helpful process, especially when they start realizing what they can do. These questions are pretty, straightforward right, they’re about your body […] there’s no right or wrong, they just have to say how they’re feeling, and I think that kinda introspection’s really important at that stressful time.*

HCP 7—Surgeon.

Finally, HCPs found that providing patients with assessments fosters a sense of hope by giving them something to look forward to completing after their surgery.

*The fact I’m talking to them about the idea of optimizing their journey through this treatment, it, I guess maybe sends a signal that we’re already thinking about that far, so it’s not all doom and gloom, it’s not like ‘I don’t even know if you’re gonna make it through this surgery.’ If I’m already talking about what comes after, then I think kind of puts a bit of a positive spin on things.*

HCP 10—Surgeon.

#### 3.2.2. Theme Two: Value of Exercise and Its Importance in Clinical Care

##### Patient Perspectives

Collectively, our sample of patients spoke to the value of exercise as a pillar in their cancer experience. While only four patients reported engaging in regular exercise, all pointed to the need for movement as an integral component of their recovery post-surgery. Specifically, patients described the value of multiphasic exercise prehabilitation at three timepoints, pre-surgery, in-hospital, and post-discharge, into recovery. Some patients spoke to the positive benefits experienced from a multiphasic exercise prehabilitation approach to date (e.g., helping prepare for surgery, helping to maintain a healthy lifestyle) and to the perceived benefits if they had more exercise support during their care (e.g., potential improvements to in-hospital recovery). Exercise in general was considered important to help patients feel better both physically (e.g., improved mobility) and mentally (e.g., more headspace). Engaging in exercise was viewed as not only important for their recovery and surviving their cancer, but for overall health status.

*It brings a normality back rather than the sick room and the hospital and sitting in a chair and trying to reach over, it brings movement back to this arm and exercise the muscles on my right shoulder and uhm, doing all those small exercises get the body moving, so I think the whole-body exercise is so important.*

P 6.

*I think the only reason why I survived the first time with stage four was because I was fit to be able to do it. How many people survive stage four? Usually that’s a death sentence.*

P 2.

##### HCP Perspectives 

As a result of seeing and hearing the benefits of exercise from their patients, HCPs described a multiphasic exercise prehabilitation approach as critical for recovery, emotional well-being, functional well-being, hospital length of stay, and wound recovery. Some HCPs spoke to the differences observed in patients who engage in exercise across their care trajectory, compared with those who do not. Collectively, HCPs felt that a multiphasic exercise prehabilitation approach should play a role in HNC surgical care, from pre-surgery to in-hospital, through immediate recovery, and into survivorship. Moreover, all HCPs indicated they were already sold on the idea of exercise for their patients.

*It’s priceless, I mean it can’t be overstated how valuable it is […] having seen so many patients over so many years, the ones that move feel better, recover better and just feel more alive, they just do better, so I think this is very, very important.*

HCP 6—Surgeon.

*Exercise is a hugely important part of going through a big surgery and hugely important to the recovery process. I’m kind of already sold on that idea, it plays a huge role and I think it’s huge psychologically and emotionally for that part of things, for people – their resilience, being able to get through the recovery and feeling stronger, and better after the fact.*

HCP 8—Surgeon.

#### 3.2.3. Theme Three: The Components of an Ideal Multiphasic Exercise Prehabilitation Program

##### Patient Perspectives

Detailed perceptions of the multiphasic exercise prehabilitation program FITT Principles are described in Table S1. There was variation in what patients preferred at each phase of their recovery. Some patients spoke to the need for a structured exercise program delivered by an exercise specialist, while other patients preferred less formal movement recommendations.

*I’d never thought about going to the gym but I’ve uhm yeah, just walking and in you know, spring summer and fall, working in the garden and other things like that, that sort of physical activity.*

P 6.

All patients agreed that engaging in movement every day is important across surgical care, with higher intensity exercise pre- and post-hospital discharge.

*I would exercise a bit every day if I could.*

P 3.

*Oh, well, pre-surgery, probably pretty intensive.*

P 3.

*In-hospital was good, I mean, if you do it for an hour in the hospital, you’re getting quite a bit of exercise in.*

P 1.

*Is this in-hospital? I’d suggest it for fifteen minutes to begin with somebody.*

P 2.

*Would be good for me in the hospital, there like, five minutes is enough.*

P 3.

*Well, I think you should start out slower and increase by the time you get to the six weeks.*

P 1.

Overall, patients spoke to the need of considering individual factors such as exercise preferences, accessibility, and other barriers and facilitators.

*Individualize it and make them feel more special.*

P 2.

Further, patients were receptive to the idea of a multiphasic exercise prehabilitation program when presented with the terms “*movement*” and “*physical activity*” instead of “*exercise*.”

*Yeah, I think movement’s a gentle way to say ‘you know, get up and move’ you know what I mean? Get up and walk, your heart doesn’t have to race at 170 beats a minute.*

P 8.

*I wouldn’t have bought-into jumping into a high intensity short-term program.*

P 10.

##### HCP Perspectives

When describing an ideal multiphasic exercise prehabilitation program, HCPs highlighted the need for exercise specialists involved in the care pathway. HCPs also felt that multiphasic exercise prehabilitation should be comprehensive and tailored to each patient, as well as indicated the importance of program timing to promote patient engagement.

*I wish we could start this tomorrow. I wish we could have an exercise physiologist in our clinic.*

HCP 2—Oncology Nurse.

*I definitely think that the patients would be pleased to hear that there’s a program in place that they have access to, and again this whole idea that to some degree, dove tails into survivorship. I definitely see this as a huge bonus, this would really upgrade what we have to offer these patients.*

HCP 10—Surgeon.

*The ideal time do present all this to the patient would be on a second visit, that comes after the initial sorta, discussion about the primary treatment […] I think that they need a bit of time to just sort of digest that, even if like, for 24hours, just to kind of get their head around what’s going on […] if there was a way to make this part of the package, to present this information like even a day later or something, the patient would absorb a lot more of this.*

HCP 10—Surgeon.

#### 3.2.4. Theme Four: Key Factors Will Support Implementation

##### Patient Perspectives

Overall, it was clear that patients value the role of exercise, with a key focus needed on implementation. Patients spoke mostly to the need for more education and access to resources relevant to their recovery. Patients were interested in education about movement recommendations pre- and post- surgery, data to support the benefits of exercise, nutritional advice, additional support for wound care/rehabilitation during immediate recovery, and more guidance for what to expect along their surgical trajectories.

*I think a quick consult before they release you from the hospital […] to sit down and say ‘okay, these are resources available and these are the things you can and can’t do,’ cause I have this new flap, am I not supposed to have a beer? What can’t I do with this flap, they never ever told me, right?*

P 8.

Patients also described the importance of having their surgeon advocate for exercise and encourage participation. While patients expressed interest in participating in a multiphasic exercise HNC surgery program, they felt that a fundamental component is receiving support from their surgeon.

*You really wanna listen to him I would think, he’s the big guy.*

P 4.

*If the doctor woulda told me ‘we need you to get your heart movin’ so get workin’ out hard here in the next month’ I would’ve worked out super hard every day before surgery, right? Uhm, but there was really nothing, just ‘go about your life’ right, ‘enjoy your life’.*

P 8.

##### HCP Perspectives

HCPs were enthusiastic about a future multiphasic exercise prehabilitation program for HNC surgical patients. Key areas for implementation included presenting data for HCPs, including more education for both patients and HCPs, realizing a culture shift in the clinical setting for cancer care (i.e., adopting the idea of multiphasic exercise prehabilitation as part of standard cancer care), as well as adapting a collaborative team approach along with continuous buy-in from the surgical team.

*I think what you’re really trying to achieve is a cultural shift, a change in brain functioning for everybody. I think what I would be looking at is what is the brand of exercise in cancer care and how do we change that on a much larger scale than largely advertising what we see in the hospital that is going out to people who already believe it.*

HCP 7—Surgeon.

Similar to patients, HCPs viewed the surgeon’s influence as essential for patient engagement with a multiphasic exercise prehabilitation program.

*I do think that it’s important that the patient’s surgeon at some point has a direct conversation with the patient to uh indicate how really important it is.*

HCP 8—Surgeon.

Several HCPs also pointed to the need of external funding and sustainability considerations for embedding a multiphasic exercise prehabilitation program outside the context of a study. A majority of HCPs perceived a number of barriers, namely insufficient funding and lack of support from administrative and policy levels.

*Right now, it’s working because it’s within the context of a study, and even more so when it becomes a [prehabilitation trial]. I would imagine there needs to be some funding to provide support, staff, and so forth to actually take the patients through all this. Like, if it becomes standard of care, who’s actually gonna administer all this stuff?*

HCP 10—Surgeon.

## 4. Discussion

The purpose of this study was to gather information from two key stakeholder groups – patients and HCPs – on adding exercise-based patient assessments across the HNC surgical timeline, and the logistics and potential benefits of a multiphasic exercise prehabilitation intervention. A key finding from this process was both patients and HCPs perceived assessments of PROs, physical function, and mobilization as important and acceptable across the HNC surgical timeline. Moreover, both groups value the idea of a multiphasic exercise prehabilitation program. Both patients and HCPs highlighted that there is limited education and support for patients and that a culture shift is needed, including at the policy level, to support adding exercise to standard cancer care. Finally, both patients and HCPs emphasized the importance of tailoring the future multiphasic exercise prehabilitation program to the unique needs of each individual patient. This study demonstrated the importance of education, attitudes, and access to resources for successful implementation of a multiphasic exercise prehabilitation program. 

Our findings are consistent with earlier work on the perceived benefits of exercise in cancer care in other tumor groups [30,31,32]. Patients scheduled for radical cystectomies had positive attitudes towards exercise prehabilitation for improving physical, emotional, and social outcomes [30]. Colorectal and ovarian cancer surgical patients also found the opportunity to engage in exercise prehabilitation as influential for recovery and distracting from negative thoughts [31]. Across this work, there are considerations for both opportunities and challenges to a multiphasic exercise prehabilitation approach (e.g., ideal timing of exercise components before acute treatment, in-hospital, and prior to or concurrent to adjuvant treatments, as well as into early survivorship phases). In line with the present findings, Payne et al., [32] pointed to the need for education for HCPs and a “*mindset change*” regarding the perceptions of exercise as a supportive cancer care resource [19,33]. 

### 4.1. Future Implications for a Multiphasic Exercise Prehabilitation Intervention

As identified in this study, an important consideration within a multiphasic exercise prehabilitation program is the FITT principle (the frequency, intensity, time, and type of exercise) and the need to tailor to patient needs and preferences at different timepoints across their cancer care trajectory. Based on participant feedback, a multiphasic exercise prehabilitation program may need to focus on “movemen*t*”, and limited exercise prescription during times of physical and psychosocial stress (pre and in-hospital phases). After hospital discharge and into immediate recovery prior to potential start of adjuvant treatment (chemotherapy and/or radiation treatment), exercise prescription can focus on immediate treatment-related side effects and focus on functional limitations. Finally, concurrent to adjuvant treatment as well as after and into survivorship, exercise prescription can begin to address core exercise principles of progression and overload, addressing treatment-related side effects, building fitness and functional benefits in patients, and enhancing overall well-being and quality of life. By “starting low and progressing slow,” exercise interventions will be more likely to facilitate behavior change and build a habit of regular movement and exercise in HNC patients, thus improving recovery. 

### 4.2. Utilizing a Patient-Oriented Research Approach 

Patient-oriented approaches have emerged with efforts to improve the credibility of research and the delivery of patient care and for recognizing the patient as the “*expert*” of their own health [34]. While a gold standard for conducting patient-oriented research does not exist, many agree that involving participants during multiple stages across the research continuum is a fundamental component [35], empowering the patient’s voice in the intimate decisions of their care [36]. Moreover, changing clinical practice is a challenge, partially due to the evidence–practice gap that often has a decade’s delay from research to implementation [37]. In an effort to bridge this gap, involving HCPs in the research process from design to implementation of new care practices is important to facilitate the rate at which knowledge is disseminated into practice [37,38]. If multiphasic exercise prehabilitation is going to be integrated into the HNC surgical care pathway, patient and HCP perspectives, as well as other critical perspectives including those of families and administration within the cancer care system will remain critical to ensure implementation efforts are successful.

### 4.3. Limitations and Future Directions

While this study adds patient and HCP perspectives on the role of exercise across clinical HNC surgical care, other perspectives of key stakeholders will be critical to support exercise implementation. For example, understanding the barriers and facilitators to exercise implementation from administrative and policy levels will support integration into clinical practice. As mentioned by one of the reconstructive surgeons (HCP 7) involved in patient care: “*I suspect there’s a whole bunch of negative perceptions around encouraging cancer patients to do exercise, so, I’m sure there’s some work that could be done on branding and marketing to make it kind of a standard that both as healthcare professionals, administrators, people financing the whole thing, tax payers, which is the public, see the importance of it***”.**

Second, our sample size of 20 allowed us to gather varied perspectives across both patients and HCPs, representing divergent (e.g., components of the FITT principle) and consistent (e.g., the importance of exercise prehabilitation) views. Due to the COVID-19 pandemic and HCPs being required on the frontlines of healthcare, we were unable to continue with further interviews. It is possible that a larger sample size may have resulted in supplementary views that would add to our understanding. 

Third, our sample of patients included mainly self-reported white males (9/10 patients), thus perspectives from other populations (e.g., ethnic minorities) were not included in this study. Further, while all surgical head and neck cancer patients were informed about the study, it is possible that those more motivated and/or keen to be involved in exercise prehabilitation chose to participate. Future work must thus focus on equity, diversity, and inclusion in exercise oncology, from considerations of recruitment into patient-oriented research work and the needs of non-English speaking participants [39] to assessment and interventions that are designed to consider ethnic and cultural needs [40].

Finally, within qualitative research, it is important to acknowledge researcher bias. The lead researcher is a white cis-gender female never having been diagnosed with cancer. Thus, to support credible research practices, the lead researcher keeps a reflexivity journal, has more than eight years working directly with patient populations in exercise oncology, and engages in ongoing qualitative training [26,41].

Despite these limitations, the current qualitative findings, in conjunction with the quantitative findings [23] support the role of a multiphasic exercise prehabilitation program across HNC clinical care. To support implementation, screening, triage, and referral pathways across the HNC continuum may support earlier access to evidence-based exercise resources for patients, as well as support better adherence to exercise programming [19,42,43,44]. In addition, supporting exercise adherence needs to extend beyond the individual. Specifically, we must consider the individual’s relationships (i.e., friends, family, healthcare providers), community (e.g., exercise oncology programs), and system (e.g., cancer care system) as components that facilitate (or deter) exercise behavior change. The Social Ecological Model [45] posits that behavior change is indeed influenced by interactions between these multiple environments. Thus, exercise oncology interventions must target multiple levels to support exercise adherence, considering interactions between patients, their healthcare providers, and the exercise specialist team, in order to build a pathway that supports a multiphasic exercise prehabilitation approach, ultimately building resources to support exercise into cancer survivorship that are sustainable in community-based settings [19,42]. To that end, tailoring interventions that focus on tumor type, treatment type, place on cancer continuum, side-effects from treatment, and individual factors is also important to enhance recruitment and adherence to exercise programming [42].

## 5. Conclusions

These qualitative findings of patient and healthcare provider perspectives on building multiphasic exercise prehabilitation into the surgical care pathway for HNC identified four themes: (1) acceptability and necessity of assessments, (2) the value of exercise, (3) the components of an ideal exercise program, and (4) factors to support implementation. Together with the quantitative findings [23], future design and implementation of multiphasic exercise prehabilitation programs for surgical HNC patients must continue to prioritize key stakeholder perspectives and the influence across multiple levels to support exercise behavior change. Our findings are currently being implemented within our multiphasic exercise prehabilitation program within the care pathway for HNC surgical patients (NCT04598087).

## Figures and Tables

**Table 1:** Baseline characteristics of participants: patients, *n* = 10 and healthcare providers, *n* = 10.

Patient Characteristics	No. of Patients (%)
**Gender** (Self-Identified) Male Female	9 (90%) 1 (10%)
Age: Mean ± SD, y	60.8 ± 8.5
**Time Until Surgery: Mean ± SD, days**	10.5 ± 8.6
**Primary Tumor Site** Oral Cavity Oropharynx Paranasal Sinuses	8 (10%) 1 (80%) 1 (10%)
Cancer Stage I II III IV Unknown	1 (10%) 2 (20%) 2 (20%) 4 (40%) 1 (10%)
**Histology** Squamous Cell Carcinoma	10 (100%)
**Ultimate Treatment** Surgery Alone Surgery + Radiation Therapy Surgery + Radiation Therapy and Chemotherapy	4 (40%) 3 (30%) 3 (30%)
Patient Demographic Variable	No. of Patients (%)
**Race (Self-Identified)** White Not Specified	9 (90%) 1 (10%)
**Employment Status** Disability Part Time Full Time Unemployed	1 (10%) 3 (30%) 4 (40%) 2 (20%)
**Annual Family Income, CDN$** $60,000–79,999 $80,000–99,000 >100,000 Prefer Not to Answer	1 (10%) 1 (10%) 3 (30%) 5 (50%)
**Smoking Status** Never Smoked Ex-Smoker Current Smoker	2 (20%) 6 (60%) 2 (20%)
**Alcohol Consumption** Never Drinker Light Drinker Moderate Drinker Heavy Drinker Previous Drinker	3 (30%) 2 (20%) 1 (10%) 2 (20%) 2 (20%)
Healthcare Providers (*n* = 10)	No. of Healthcare Providers (%)
**Gender** (Self-Identified) Male Female	4 (40%) 6 (60%)
**Discipline** Surgeon Oncology Nurse Physiotherapist Unit Manager Clinical Nurse Educator Unit nurse/research assistant	4 (40%) 2 (20%) 1 (10%) 1 (10%) 1 (10%) 1 (10%)

## Data Availability

Study interview guides can be found online (https://osf.io/qw4zx/ accessed on 12 July 2022).

## Supplementary Materials

The following supporting information can be downloaded at: https://www.mdpi.com/article/10.3390/curroncol29080469/s1, Document S1: Interview Guides; Table S1: Patient Perspectives of Multiphasic Exercise Prehabilitation Program Characteristics: Frequency, Intensity, Time, and Type (FITT) Principles - Additional Quotes.
