# Supplementary material for: A Qualitative Study of Patient and Healthcare Provider Perspectives on Building Multiphasic Exercise Prehabilitation into the Surgical Care Pathway for Head and Neck Cancer
Source: Curr Oncol. 2022 Aug 21;29(8):5942–54. doi: 10.3390/curroncol29080469 (PMC9406549; doi:10.3390/curroncol29080469)
Supplement: Supplementary file 1 [file curroncol-29-00469-s001.zip › curroncol-1849042-supplementary-final/curroncol-1849042-supplementary#1.pdf]

Head and Neck Cancer Feasibility Study

Interview Guide #1 – Patient

<u>Location:</u> *In hospital, prior to discharge*

SCRIPT

WELCOME

Hello ______. Thank you for taking the time to meet with me today. Before we get started with the interview, I would like to go over some of the details.

PURPOSE

The purpose of today's interview is to get your perspective on the acceptability of the measures we completed throughout your surgical timeline.

CONSENT PROCESS

Please take a moment to review this form. It has an overview of today's process, as well as some important considerations related to your privacy and comfort. As a reminder, your anonymity and confidentially will be kept throughout the entire research process. The data from today will be stored in a confidential, secure location in the Health and Wellness Lab, University of Calgary. This interview should take about 30-45 minutes to complete. Should you need a break, we can stop at any time. You also have the ability to withdraw at any point from the interview. I have some questions prepared, but if you wish not to comment on something, that is perfectly fine. Also, please keep in mind that there are no right or wrong answers. I will be recording our interview, which will be placed on the table, as well as possibly taking notes periodically. Do you have any questions? Ready?

QUESTIONS

---

1. **I'd like to begin by asking about your overall experience with your surgical treatment thus far?**
   Probes:
   - *And why would you say that?*
   - *Can you think of an example?*

2. **Could you please tell me about how you have felt about your assessments so far?**
   Probes:
   - *Ease/difficulties?*

3. **If you remember, you were introduced to this study a few weeks before your surgery, and a member of the research team contacted you to further explain what it would entail. Could you please tell me about your experience with this recruitment process?**
   Probes:
   - *How was the timing?*

4. **We had a number of assessments. I am going to ask you about each one.**

   A: Could you please tell me how you found the questionnaires?
   Probes:
   - *Were they manageable?*
   - *Anything else you'd like to tell us/have us assess?*
   - *Things we should not ask about?*

   B: Could you please tell me how you found the physical tests?
   Probes:
   - *Were they manageable?*
   - *Timing?*
   - *Duration?*

   C: Could you please tell me how you found the usage of the Garmin watch after your surgery/in hospital?
   Probes:
   - *Was it useful?*
   - *Did you refer to it during the day?*
   - *Did your nurse/HCP ask about it?*

5. **To date, we completed these assessments at two timepoints: before and after surgery. Could you please tell me how you felt about the timing of the one before your surgery at the University of Calgary and the one after surgery, in hospital?**

Probes:

- *Was it manageable?*
- *Do you have any examples of what you've liked or found difficult with these timepoints?*
- *Duration?*

6. **Could please tell me if there is anything about the assessments that you'd like to see changed?**

   Probes:

   - *ONLY ask if not covered in previous answers*

7. **Is there anything else you would like to tell me about your experience in this study?**

Thank you very much for participating in today's interview. Please do not hesitate to contact me if you have any outstanding questions.

Head and Neck Cancer Feasibility Study

Interview Guide #2 – Patient

Location: *University of Calgary; 6 ±2-weeks post-surgery* OR *Remote*

QUESTIONS

I have a number of questions prepared for us today and am looking to get your honest feedback.
In the first interview, we asked about your participation in the assessments. Now we are interested in getting your feedback about how we can build an exercise program for surgical head and neck cancer patients into the care pathway.

1. **I'd like to begin by asking about your overall experience with the assessments and use of the Garmin watch in hospital?**
   Probes:
   - *Can you tell me more?*

2. **Could you please tell me about your physical activities before and after your cancer diagnosis?**
   Probes:
   - *What has the impact of the treatment been on your activity level?*
   - *Do you see a role of activity in your recovery?*

3. **We know that exercise is beneficial to cancer patients during the cancer continuum, but it is still not included in usual care. Given that you've just gone through this experience, we want to know your opinion of what an exercise program could look like.**

   A: I'm going to give you a potential scenario for what an exercise program might look like, then ask you some questions:

   You meet with your doctor to discuss your surgical treatment, and you are provided with the following program:
   - Before and after surgery assessments (questionnaires, tests of physical function)
   - A Garmin watch to use from diagnosis through recovery (to about this timepoint that we are now meeting)
   - An individualized exercise program for pre-surgery, in-hospital, and after surgery through immediate recovery up to about 6 weeks post.
   - Transition into a 12-week community-based exercise program

B: Could you please tell me about how often you think you might like to engage in exercise during each of these phases:

Pre-surgery
In-hospital
Post-surgery up to about 6 weeks after

C: Could you please tell me what intensity level you think you might be able to work at?

Pre-surgery
In-hospital
Post-surgery up to about 6 weeks after

D: Could you please tell me how long you think you might like these exercise sessions to last?

Pre-surgery
In-hospital
Post-surgery up to about 6 weeks after

E: Could you please tell me what type of exercise you think you might like to engage in?

Pre-surgery
In-hospital
Post-surgery up to about 6 weeks after

F: Could you please tell me where you think you might like to exercise?

Pre-surgery
Post-surgery up to about 6 weeks after

Probes:

- *Why do you think that?*

4. **Is there anything else you would like to tell me about your perception of a future exercise intervention like the one we just discussed?**

Probes:

- *Anything you wouldn't want in a program? Timing?*
- *Definitely want in a program?*

Thank you very much for participating in today's interview. Please do not hesitate to contact me if you have any outstanding questions. Have a wonderful day!

Head and Neck Cancer Feasibility Study

Interview Guide #3 – HCPs

<u>Location:</u> In hospital <u>OR</u> Remote

SCRIPT

WELCOME

Hello ______. Thank you for taking the time to meet with me today. Before we get started with the interview, I would like to go over some of the details for today.

PURPOSE

The purpose of today's interview is to get your perspective on the acceptability of measuring patient-reported outcomes, physical function, and in-hospital mobilization throughout the surgical timeline in head and neck cancer patients. This means I will be asking you questions related to your experience with the study so far.

CONSENT PROCESS

Please take a moment to review this form. It has an overview of today's process, as well as some important considerations related to your privacy and comfort. As a reminder, your anonymity and confidentially will be kept throughout the entire research process. The data from today will be stored in a confidential, secure file at the Health and Wellness Lab, University of Calgary. This interview should take about 30-45 minutes to complete. Should you need a break, we can stop at any time. You also have the ability to withdraw at any point from the interview. I have some questions prepared for today, but if you wish not to comment on something, that is perfectly fine. Also, please keep in mind that there are no right or wrong

answers. I will be recording our interview with a recorder, which will be placed on the table, as well as possibly taking notes periodically. Do you have any questions?

QUESTIONS

---

1. **First, I'd like to ask about your health care provider role. What is your interaction with patients and other health care professionals?**
   Probes:
   - *Years of experience*

2. **Could you tell me about your overall experience with how we have assessed the patient-reported outcomes and fitness measures surgical head and neck cancer patients?**
   Probes:
   - *Overall, impact on your HCP role?*

3. **Could you please tell me about what you think about adding in these measures to the usual care for the HNC surgical patients**
   Probes:
   - *Do you think it is worthwhile?*
   - *Do you have any examples of what you've liked or found difficult with the study?*

4. **To recruit for this study, patients were approached at the time of surgical consent and were given a 'consent-to-contact' form. Next, a member of our study team contacted them to explain our study. Could you please tell me about your experience with this recruitment process?**
   Probes:
   - *Do you have any examples of what you've liked or found difficult?*

5. **A research assistant visited the ward to collect data of physical function tests of patients after their surgery. Could you please tell me how you found this process?**
   Probes:
   - *Did this cause you any problems? Impact your role?*
   - *What impact of timing of the assessments for your role?*
   - *Did you find them acceptable?*
   - *Was it burdensome/ difficult to carry out?*
   - *Was it manageable?*
   - *Do you have any examples of what you've liked or found difficult?*

6. **Could you please tell me how you found the usage and assistance of the Garmin watch during the in-hospital time?**
   <u>*Probes:*</u>
   - *Did you find it acceptable? Useful? Did you provide feedback to the patient based on the Garmin watch?*

7. **Could you please tell me about your thoughts on how the patients experienced the online questionnaires?**
   <u>*Probes:*</u>
   - *Did the patients mention anything, positive or negative, about the questionnaires?*
   - *Did you notice any positive/negative impact?*

8. **Could please tell me if there is anything about the study you liked/didn't like as we consider implementing this into the care pathway?**

9. **Given your experience with the study so far, could you please tell me about your perception of the following hypothetical situation:**
   A head and neck cancer patient meets with their doctor to discuss surgical treatment, and is provided with the following program:
   - Before and after surgery assessments (questionnaires, tests of physical function)
   - A Garmin watch to use from diagnosis through about 6 weeks after surgery
   - An after-surgery individualized exercise program, which would include a 30-60-minute session with an exercise specialist, in hospital, tailored to your specific needs and abilities
   - A free, 12-week community-based exercise program post hospital discharge (ACE)

10. **If we were to make this study part of usual care, what would be your thoughts on the benefits of exercise for head and neck cancer patients?**
    <u>*Probes:*</u>
    - *How much do you know about the benefits?*
    - *Would education for you be useful in your role?*
    - *What would you like to learn?*

11. **Is there anything else you would like to tell me about your experience in this study?**

Thank you very much for participating in today's interview. Please do not hesitate to contact me if you have any outstanding questions.

Head & Neck Cancer Feasibility Study

Interview Guide #3 – HCPs (Surgeon)

<u>Location:</u> *Remote*

SCRIPT

WELCOME

Hello ______. Thank you for taking the time to talk with me today. Before we get started with the interview, I would like to go over some the details.

PURPOSE

The purpose of today's interview is to get your perspective on the acceptability of measuring patient-reported outcomes, physical function, and in-hospital mobilization throughout the surgical timeline in head and neck cancer patients. I will be asking you questions related to your experience with the study so far.

CONSENT PROCESS

As a reminder, your anonymity and confidentially will be kept throughout the entire research process. The data from today will be stored in a confidential, secure file at the Health and Wellness Lab, University of Calgary. This interview may take 15-30 minutes to complete. You also have the ability to withdraw at any point from the interview. I have some questions prepared for today, if you wish to add something, please jump in. I will be recording our interview with a recorder, as well as taking notes. Do you have any questions?

QUESTIONS

---

1. **What was your involvement with this study?**

2. **To recruit for this study, patients were approached at the time of surgical consent and were given a 'consent-to-contact' form. Next, a member of our study team contacted them to explain our study. Could you please tell me about your experience with this recruitment process? Use their recruitment numbers and ask about that flow in their usual work (use surgeon's recruitment numbers**)

   Probes:

- *Do you have any examples of what you've liked or found difficult?*
- *Impact on workflow?*
- *Is there anything you would like to see changed with this process?*
- *How did you find the timing?*
- *Role of supporting staff?*

3. **if you had experience with the Patient reported outcomes and fitness measures, can you tell me what it was like?**

Probes:

- *Overall, impact on your HCP role?*
- *Perceived value of this?*

4. **Could you please tell me about what you think about adding in these measures to the usual care for the HNC surgical patients?**

   Probes:

- *Do you think it is worthwhile?*
- *Do you have any examples of what you've liked or found difficult with the study?*

5. **A research assistant visited the ward to collect data of physical function tests of patients after their surgery. Were you aware? Did your patients ask you questions?**

Probes:

- *Did this cause you any problems? Impact your role?*
- *What impact of timing of the assessments for your role?*
- *Did you find them acceptable?*
- *Was it burdensome/ difficult to carry out?*
- *Was it manageable?*

- *Do you have any examples of what you've liked or found difficult?*

6. **Could you please tell me how you found the usage and assistance of the Garmin watch during the in-hospital time?**
   Probes:

- *Did you find it acceptable? Useful? Did you provide feedback to the patient based on the Garmin?*

7. **Could you please tell me about your thoughts on how the patients experienced the online questionnaires?**

Probes:

For questions 5-8 – based on their experience as a clinician, value they see in all of these being added to usual care of these surgical patients

- *Did the patients mention anything, positive or negative, about the questionnaires?*
- *Did you notice any positive/negative impact?*

8. **Given your experience with the study so far, could you please tell me about your perception of the following hypothetical situation:**
   A head and neck cancer patient meets with you to discuss and consent to their surgical treatment. Next, they are provided with the following program:
   - Before and after surgery assessments (questionnaires, tests of physical function)
   - A Garmin watch to use from diagnosis through about 6 weeks after surgery
   - An after-surgery individualized exercise program, which would include a 30-60-minute session with an exercise specialist, in hospital, tailored to your specific needs and abilities
   - A free, 12-week community-based exercise program post hospital discharge (ACE)

9. **What do you need from us, as exercise experts, to make this successful within the hospital setting/standard surgical care timeline?**

10. **Is there anything else you would like to tell me about your experience in this study?**

Thank you very much for participating in today's interview. Please do not hesitate to contact me if you have any outstanding questions.
