# Supplementary material for: A Qualitative Study of Patient and Healthcare Provider Perspectives on Building Multiphasic Exercise Prehabilitation into the Surgical Care Pathway for Head and Neck Cancer
Source: Curr Oncol. 2022 Aug 21;29(8):5942–54. doi: 10.3390/curroncol29080469 (PMC9406549; doi:10.3390/curroncol29080469)
Supplement: Supplementary file 1 [file curroncol-29-00469-s001.zip › curroncol-1849042-supplementary-final/curroncol-1849042-supplementary#2.pdf]

Supplementary Materials: A Qualitative Study of Patient and Healthcare Provider Perspectives on Building Multiphasic Exercise Prehabilitation into the Surgical Care Pathway for Head and Neck Cancer

Table S1. Patient perspectives of multiphasic exercise prehabilitation program characteristics: frequency, intensity, time, and type (FITT) principles – additional quotes.

FITT PRINCIPLE	PRE-SURGERY	IN-HOSPITAL	6-WEEKS POST-SURGERY
FREQUENCY	*Pre-surgery, well I did it all the time, it was, like my daily routine.* P 1.<br><br>*Well, I was [going to the gym] three to four times a week.* P 2.<br><br>*Uhm, you know, prior to surgery I was if possible, walking every day.* P 6.	*Oh, you know, once a day would be great, just to come in and do a few movements and exercises and just keep things going, that would be good one, yeah.* P 1.<br><br>*Oh, I'd do it every day.* P 2.<br><br>*Uhm, so pretty much every day.* P 8.	*Oh, every day, yeah, something every day.* P 6.<br><br>*I would say every other day, uhm, for sure […] I guess if you're doing different activities, you could convince yourself to do something every day […] I kinda focus on my physio stuff every day, and then I do a big thing every other day.* P 10.
INTENSITY	*Eight or nine, yeah [referring to an intensity scale of 0-10].* P 1.<br><br>*Hopefully a hundred percent ha, ha, ha.* P 4.<br><br>*For me, well, yeah, I mean for me I could've handled a, a moderate uhm, I don't know if that goes for everyone, uh, but yeah.* P 10.	*Oh, maybe a five [referring to an intensity scale of 0-10].* P 1.<br><br>*In-hospital, it would be have to be say low.* P 2.<br><br>*[…] the last two days I was walkin' fairly well and everything, so I don't know, let's put that in the middle, I'll say 5 [referring to an intensity scale of 0-10].* P 3.<br><br>*Probably nothing, ha, ha, ha. It was pretty tough there, it was. I wasn't getting any getting sleep, so was, I was always tired, I could work for a little bit I guess.* P 4.	*Oh, yeah, I'd like to start out lower and move up if I could [referring to an intensity scale of 0-10].* P 1.<br><br>*Now I'm gonna be down low-moderate.* P 2.<br><br>*Uhm, I could go pretty good, I could say 7 or whatever [referring to an intensity scale of 0-10].* P 3.
TIME	*Pre-surgery, hmm, I went all day long, though, that's what I do.* P 1.	*Well, I would work as, as much as I could.* P 4.<br><br>*If you're doing exercise, consistency is better.* P 4.	*And now, like I said, I, you get around pretty good, I'd probably do easily a fifteen, twenty-minute workout.* P 3.

	*Uhm, before surgery, uhm, probably a good half an hour a day***.** P 3.<br><br>*I would say a simple, something simple, something doable, maybe fifteen to half an hour a day, I probably could've managed***.** P 10.	*Couple of [my walks] I'd go out to the [the surgical building] and back three or four times, so they were twenty minutes, half an hour.* P 6.	
**TYPE**	*I like to do all my exercises.* P 1.<br><br>*Well, probably what I would and my figure out gonna be possibly dealing with uh, strengthening the leg cause I knew that they were gonna take whatever out of the leg and everything, so that's mainly what I was uh, thinking of is the legs, you know.* P 3.<br><br>*I would say simple things from your home uhm, would be achievable for everyone […] certainly stretching, uhm, you know, sort of that general, just like you had mentioned, like breathing or some just simple yoga, stretches.* P 10.	*Oh I'd like to do the lifting and try and move my leg more and do a little walking, yeah, and core strength.* P 1.<br><br>*Uhm, walking to the washroom, yeah, walking.* P 3.<br><br>*Oh, whatever. They are the specialist [referring to the type of exercise an exercise specialist could deliver in-hospital].* P 4.<br><br>*[…] if the exercise involves laying on the ground and moving one's arms, that's not possible in-hospital.* P 6.<br><br>*Discussion and demo and trial but then I could see like, going to physio afterwards maybe a visit to make sure everything is being done correctly, cause I know you can damage yourself if you do uh, exercise incorrectly.* P 6.<br><br>*I mean certainly walking uhm, I you know I could've, I mean those sit-to-stand squats certainly were feasible uhm, you know, I couldn't have gotten on the floor to do anything […] I don't know that I'd get back up uhm, or would want to be on the floor.* P 10.	*Well, post-surgery, you wanna be do somethin' just to keep your strength up and you know I'd like to try and increase a little bit all the time to get back to being normal.* P 1.<br><br>*The post-surgery afterwards you know, is nice to have a bit of a program to follow so that you know my leg gets better and I'm doing the things that I should do and raisin my arms and stuff like that.* P 1.<br><br>[P 2 likes to follow a structured resistance training program].<br><br>*I would like to have a program. I'm doing a little bit now […] they give me one or two exercises and they'll see you in a month.* P 4.<br><br>*[…] I like walking, I've walked all my life, I would uh like to bike when I'm not there physically yet uhm, so if there are other exercises or ways I could get uhm, get my body back uh, faster that would be better, especially if radiation is uh in the books, then, get back to as fit as possible before radiation starts and […] uhm, the body sort of deteriorates during radiation and due to exhaustion.* P 6.

			[P 7 preferred home-based activities of daily living].<br><br>*Uh, mostly running, I think, uh I do have free weights at home, so, so I may hit weights again.* P 8.
**LOCATION**	*Oh, I stay home, I like that, never been much for the gym.* P 1.<br><br>*I can see myself committing to doing exercises at home on my own, but I can't see myself committing to go out to somewhere and do it.* P 3.<br><br>*Probably in a group and by myself.* P 4.<br><br>*I personally don't like group-based so it'd probably be better because then there's more pressure to push yourself harder, it's easiest to not push yourself when you're alone in your home.* P 6.<br><br>*I would not have made the time to go somewhere.* P 10.	N/A<br>[all would take place in-hospital]	*I would do it in the home in more structured so that you're doing what you're supposed to do.* P 1.<br><br>[P 2 preferred exercising in a gym].<br><br>*I'd do it at home, you know probably, I just can't see myself going out to a fitness centre*. P 3.
